# Variation of insertion of the pectoralis major in a cadaveric study: A case report

**DOI:** 10.1097/MD.0000000000021475

**Published:** 2020-07-31

**Authors:** Shuji Katsuki, Hayato Terayama, Ryuta Tanaka, Ning Qu, Hayato Nomura, Satoshi Kawakami, Kanae Umemoto, Kaori Suyama, Shuang-Qin Yi, Takeshi Suzuki, Kou Sakabe

**Affiliations:** a Department of Rehabilitation, Kanto Rosai Hospital, Nakahara-ku, Kawasaki-si; b Department of Anatomy; c Department of Anesthesiology, Tokai University School of Medicine; d Department of Public Health, Tokai University School of Medicine and Nursing, Isehara-si, Kanagawa, Japan; e Department of Frontier Health Sciences, Graduate School of Human Health Sciences, Tokyo Metropolitan University, Arakawa-ku, Tokyo, Japan.

**Keywords:** bicipital tunnel, cadaver, insertion, long head of the biceps brachii, pectoralis major

## Abstract

**Rationale:**

Typically, the tendon of the pectoralis major inserts into the crest of the greater tubercle of the humerus. However, anomalous insertion sites of the pectoralis major tendons have been noted.

**Patient concerns:**

The cadaver of a 95-year-old Japanese man was selected from the bodies used for gross anatomy practice at the Tokai University School of Medicine in 2018.

**Diagnosis:**

In this cadaver, the left side of the pectoralis major tendon appeared to insert at the crest of the greater tubercle and lesser tubercle of the humerus, forming a tunnel measuring 2.5 cm in total length.

**Intervention:**

We removed the fat and skin around the shoulder joint and upper extremity for observational purposes and carefully examined the structures during gross anatomy.

**Outcomes:**

The medial side of the insertion of the pectoralis major tendon was not into the humerus but had combined with the tendon of the latissimus dorsi, which then loosely inserted into the humerus. As the roof and both walls comprised the tendon of the pectoralis major and the floor was formed by the tendon of the latissimus dorsi and humerus, the structure formed a tunnel.

**Lessons:**

This study is important for orthopedic and rehabilitation physicians in treating diseases of the long head of the biceps brachii tendon. As part of management, the condition of the tendon of the pectoralis major should be confirmed using magnetic resonance imaging or echocardiography.

## 1 Introduction

**Figure 1:**
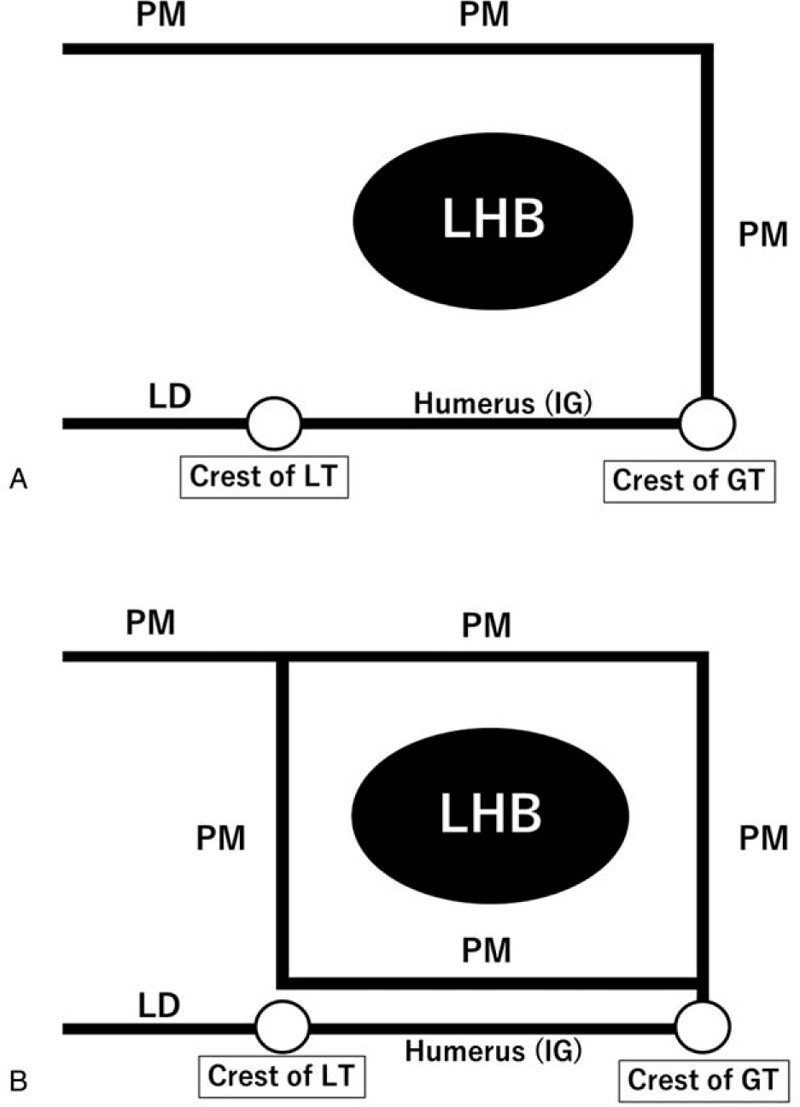
Structure of the pectoralis major tendon tunnel. (A) In the normal case, the structure of the tunnel is formed by the roof made by the tendon of the pectoralis major, and the floor formed is formed by the latissimus dorsi and humerus. (B) In Kawakami's case, the tendon of the pectoralis major formed the roof and floor of the tunnel. GT = greater tubercle, IG = intertubercular groove, LD = latissimus dorsi, LHB = long head of biceps brachii, LT = lesser tubercle, PM = pectoralis major.

In general, the pectoralis major muscle originates from the medial end of the clavicle, sternum, the second to sixth ribs, and the external oblique muscle fascia, passes through the ventral side of the long head of the biceps brachii (LHB), and inserts as one tendon into the crest of greater tubercle.^[1–5]^ The LHB tendon arises from the supraglenoid tubercle in the shoulder joint and passes through the intertubercular groove (IG) of the humerus. The latissimus dorsi muscle arises from the spinous process and iliac crest and inserts as one tendon into the crest of lesser tubercle. Therefore, around the IG, the roof is formed by the tendon of the pectoralis major, and the floor is formed by the latissimus dorsi muscle and humerus in normal cases (Fig. 1A).^[5]^ To our knowledge, anatomical studies of the insertion of the pectoralis major have so far only reported on anomalies of insertion into the crest of the greater tubercle of the humerus. However, in the present case, the tendon of the pectoralis major appeared to insert into the crest of the greater tubercle and the crest of the lesser tubercle, and a tunnel was being formed by the insertion of the pectoralis major. The tendon of the LHB adhered to this tunnel. The purpose of this report is to discuss an anomaly observed in the insertion of the tendon of the pectoralis major at the shoulder joint and to present the degenerative changes seen in the LHB.

## 2 Methods

This case utilized the cadaver of a 95-year-old Japanese man (number: 2026, cause of death: hepatic cancer) that was selected from the bodies used for gross anatomy practice at the Tokai University School of Medicine in 2018. The cadaver was fixed using 10% formaldehyde. Gross dissection was performed using standard technique.

We removed the fat and skin around the chest and both shoulder joints for observational purposes and carefully examined the structures. The anatomical relationship between the pectoralis major and the LHB was specifically observed.

This case report complies with the research guidelines of the Japanese Association of Anatomists. A cadaver designated (Tokai Daigaku Kentai No Kai) for education or research was utilized in this study. Informed consent was obtained from the antemortem person by Tokai Daigaku Kentai No Kai.

## 3 Case report

**Figure 2:**
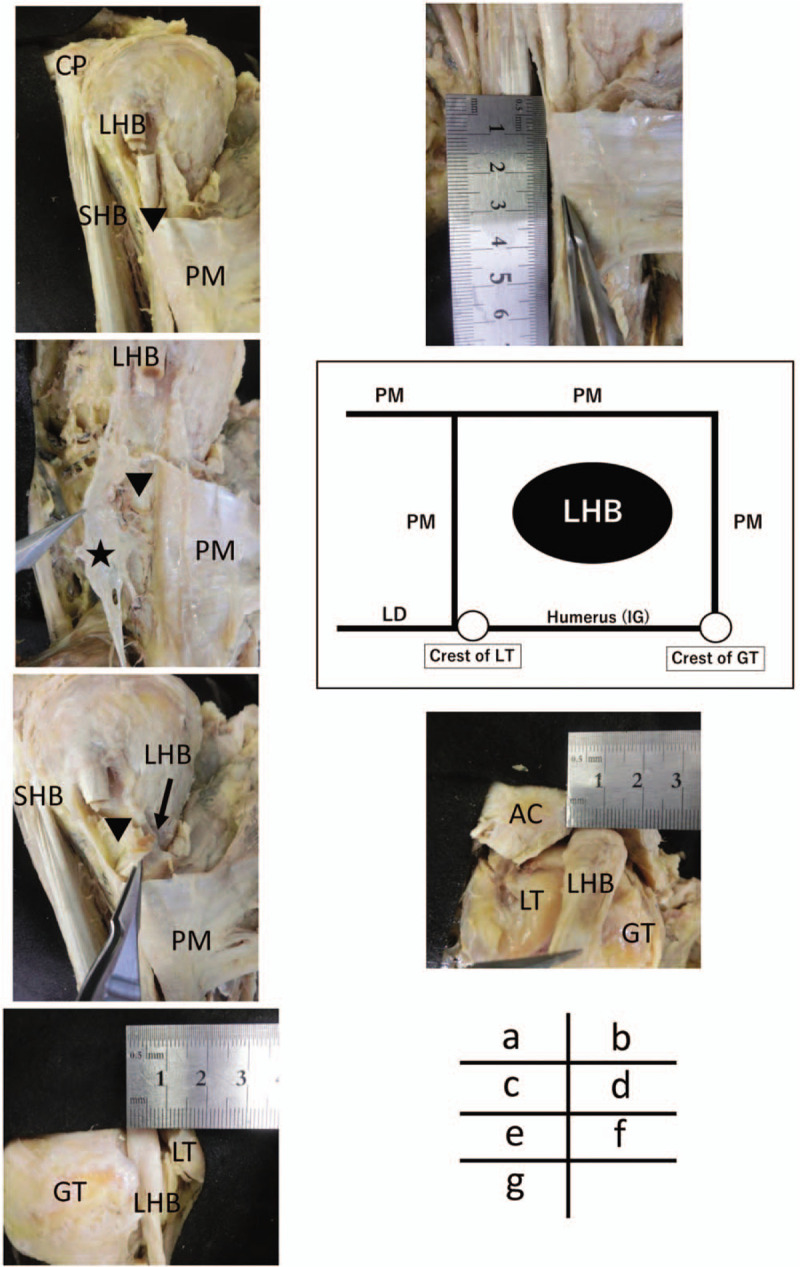
Anatomical view of the pectoralis major tendon tunnel of the left upper arm. (A) The PM tendon appears to insert at the crest of the greater tubercle and the lesser tubercle of the humerus, and the tunnel is formed by the tendon of the PM 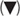. (B) The total length of the pectoralis major tendon tunnel is about 2.5 cm. (C) Medial side of the insertion of the tendon of PM 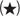 is not into the humerus, but loosely combines with the tendon of LD 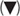. (D) The roof and both walls are formed by the tendon of the pectoralis major, and the floor is formed by the tendon of the LD loosely and humerus in our case. (E) The LHB tendon is adhered in the tunnel of the PM 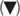. (F) The left-sided LHB tendon appears to have degenerative changes intra-articularly at gross anatomy, flattening the LHB as much as 15 mm. (G) The right side of the LHB tendon of intra-articular appears normal, flattening the LHB as much as 8 mm. AC = acromion, CP = coracoid process, GT = greater tubercle, LHB = long head of biceps brachii, LT = lesser tubercle, PM = pectoralis major, SHB = short head of biceps brachii.

In our case, the left pectoralis major tendon appeared to insert at the crest of the greater tubercle and lesser tubercle of the humerus, forming a tunnel (Fig. 2A). The total length of tunnel was about 2.5 cm (Fig. 2B). At the lateral side of the LHB, the pectoralis major tendon was inserted into the crest of the greater tubercle of the humerus. However, at the medial side of the LHB, the pectoralis major tendon combined loosely with the tendon of the latissimus dorsi and was not inserted to the crest of the lesser tubercle of the humerus (Fig. 2C). Therefore, in our case, the roof and both walls were formed by the tendon of the pectoralis major, and the floor was formed by the tendon of the latissimus dorsi and humerus (Fig. 2D). In addition, the LHB tendon was adherent in the tunnel of the pectoralis major, interiorly (Fig. 2E), and the left LHB tendon showed more intra-articular degenerative changes at gross anatomy than the right LHB tendon. Additionally, the major axis of the LHB tendon was flattened to 15 mm (Fig. 2F). The right pectoralis major was inserted into the crest of greater tubercle only and was normal. There was no pectoralis major tunnel, the LHB tendon was normal intra-articularly, and it was flattened to 8 mm (Fig. 2G).

## 4 Discussion

Typically, the tendon of the pectoralis major is inserted into the crest of greater tubercle. There are many reports on anomalies of the pectoralis major based on the pectoralis quartus variants,^[6–8]^ and the footprint of the pectoralis major tendon.^[2,3]^ However, there are few reports on the anomalous insertion of the pectoralis major tendon.^[5,9]^ In our case, the left pectoralis major tendon appeared to insert into the crest of greater tubercle and lesser tubercle, and only the pectoralis major formed a tunnel roof. Moreover, the pectoralis major tendon combined with the latissimus dorsi tendon at the medial side of the lesser tubercle.

Samuel et al^[5]^ reported that the bicipital tunnel is the extra-articular, fibro-osseous structure that encloses the LHB tendon in a normal case (Fig. 1A). The structure of the bicipital tunnel is as follows: the tendon of the pectoralis major form the roof, and the latissimus dorsi and humerus forms the floor. In another study, Kawakami et al^[9]^ reported that only the tendon of the pectoralis major formed the tunnel. His report suggested that the pectoralis major insertion was at the crest of greater tubercle, and the tendon of the pectoralis major runs beyond and behind the LHB. Hence, the structure of the tunnel was formed by the tendon of the pectoralis major making a roof and a floor (Fig. 1B). However, in our case, the structure of the roof and the lateral side was formed by the tendon of the pectoralis major inserted into the crest of greater tubercle. In addition, the medial side was formed by the tendon of the pectoralis major loosely combined with the tendon of latissimus dorsi at the medial crest of the lesser tubercle (Fig. 2D). The structure of the floor was the IG of the humerus and latissimus dorsi. Therefore, to the best of our knowledge, our case potentially describes the structure of a new pectoralis major tendon tunnel.

There is no clear opinion of the width of the LHB. Burkhead et al^[10]^ reported that the width of the normal LHB intra-articularly was 6 to 7 mm. Yoshikawa et al^[11]^ suggested that all of the flattened LHB muscles in rotator cuff tears showed dense connective tissue with some degenerative changes which included failure of the collagen bundle, hyalinization, and myxoid changes. In our case, on the right side, the width of the LHB intra-articularly was 8 mm. Hence, it appeared normal. However, on the left side, the width was about 15 mm, nearly double the width on the right. Therefore, it appeared to have sustained degenerative damage.

In this case, the tunnel was composed of the tendons of pectoralis major, similar to the sheath of the LHB tendon. Therefore, as in our case, the presence of the pectoral tendon tunnel on the outside of the shoulder joint may contribute to the stability of the LHB. However, it is possible that the LHB tendon may cause excessive friction in the pectoralis major muscle tunnel by some cause. In addition, the LHB tendon may adhere to the tunnel due to degenerative changes and it may be the limiting factor of shoulder motion.

No reports exist regarding the relationship between degenerative changes of the LHB and the structure of the tendon of the pectoralis major. Therefore, further research on this topic is warranted. Our study is important for orthopedic and rehabilitation physicians to treat the diseases of the LHB tendon. During the course of management, the condition of the tendon of the pectoralis major should be confirmed using magnetic resonance imaging or echocardiography.

## Acknowledgments

The authors thank Dr. Osamu Tanaka, Mr. Noriyuki Kosemura, Ms. Kyoko Endo, and Ms. Yuko Furuya (of Tokai University School of Medicine, Kanagawa, Japan) for excellent secretarial support. The authors also thank Editage (www.editage.jp) for aiding with English language editing.

## Author contributions

**Conceptualization:** Shuji Katsuki, Hayato Terayama, Ryuta Tanaka.

**Investigation:** Shuji Katsuki, Ryuta Tanaka, Kanae Umemoto.

**Methodology:** Shuji Katsuki, Hayato Terayama.

**Data curation:** Hayato Nomura, Kaori Suyama.

**Project administration:** Kou Sakabe, Hayato Terayama.

**Software:** Shuji Katsuki, Hayato Terayama, Satoshi Kawakami, Shuang-Qin Yi.

**Supervision:** Kou Sakabe, Takeshi Suzuki.

**Writing – original draft:** Shuji Katsuki, Hayato Terayama.

**Writing – review & editing:** Hayato Terayama, Ning Qu, Shuang-Qin Yi, Kou Sakabe.
